# Risk factors of local recurrence following implant-based breast reconstruction in breast cancer patients

**DOI:** 10.1186/s12905-021-01287-4

**Published:** 2021-04-10

**Authors:** Miwa Fujihara, Rie Yamasaki, Mitsuya Ito, Tadahiko Shien, Reina Maeda, Takanori Kin, Ayako Ueno, Yukiko Kajiwara, Kensuke Kawasaki, Kouichi Ichimura, Hiroya Mihara, Naritaka Kimura, Shoichiro Ohtani

**Affiliations:** 1 Department of Breast Surgery, Hiroshima City Hospital, 7-33 Moto-machi, Naka-ku, Hiroshima, 730-8518 Japan; 2 Department of Pathology, Hiroshima City Hospital, 7-33 Moto-machi, Naka-ku, Hiroshima, 730-8518 Japan; 3 Department of Breast and Endocrine Surgery, Okayama University Hospital, 2-5-1 Shikata-cho, Kita-ku, Okayama, 700-8558 Japan; 4 Department of Plastic Surgery, Hiroshima City Hospital, 7-33 Moto-machi, Naka-ku, Hiroshima, 730-8518 Japan

**Keywords:** Breast cancer, Immediate breast reconstruction, Implant-based breast reconstruction, Local recurrence, Risk factors

## Abstract

**Background:**

The number of patients desiring implant-based breast reconstruction has been increasing. While local recurrence is observed in patients with breast reconstruction, only a few reports have focused on the risk factors for local recurrence and the prognosis after developing local recurrence.

**Methods:**

We analyzed 387 patients who underwent implant-based breast reconstruction during the period from 2004 to 2017 in Hiroshima City Hospital. We retrospectively examined the risk factors for local recurrence and the outcomes of patients developing such recurrence after implant-based breast reconstruction.

**Results:**

The median follow-up time was 59 months. The local recurrence rate was 3.1% (n = 12). The most common reason for detecting local recurrence was a palpable mass. Four patients with local recurrence had recurrence involving the skin just above the primary lesion and needle biopsy tract. All patients with local recurrence received surgery and systemic therapy and most patients received radiation therapy, all have remained free of new recurrence to date. Multivariate analysis showed lymphatic vessel invasion (HR, 6.63; 95% CI, 1.40–31.36; *p* = 0.017) and positive or < 2 mm vertical margin (HR, 9.72; 95%CI, 1.23–77.13; *p* = 0.047) to be associated with significantly increased risk of local recurrence.

**Conclusions:**

The risk factors for local recurrence following implant-based breast reconstruction were lymphatic vessel invasion and positive or < 2 mm vertical margin. Removal of the skin just above the primary lesion and needle biopsy tract and adjuvant radiation therapy might improve local outcomes. Patients with local recurrence following implant-based breast reconstruction appear to have good outcomes with appropriate treatment.

## Introduction

Immediate breast reconstruction (IBR) following mastectomy is widely performed as part of the current breast cancer treatment in Japan, and good esthetic outcomes are obtained. There are two IBR methods; autologous tissue-based and implant-based breast reconstruction. Implant-based breast reconstruction is performed more often than autologous tissue-based breast reconstruction and is particularly preferred by younger patients with early stage breast cancer because the former is less surgically invasive than the latter approach. Moreover, we can perform implant-based reconstruction for patients who do not have sufficient autologous tissue, such that autologous tissue-based breast reconstruction is not feasible.

There is reportedly no increase in local recurrence due to the breast reconstruction procedure itself, regardless of whether the breast reconstruction is the autologous tissue or the implant-based type [1, 2]. Recently, however, local recurrence has increasingly been observed in patients with implant-based breast reconstruction in our hospital. Once local recurrence develops, combined modality therapeutic regimens are considered, including surgery, radiation, and systemic treatments, such as chemotherapy and hormonal therapy. To perform implant-based breast reconstruction that is both safe and provides a good esthetic outcome, it is important to determine the risk factors for local recurrence and the prognosis of patients with this form of recurrence. However, there are only a few reports on local recurrence following reconstruction. In this study, we retrospectively examined the risk factors for local recurrence and the prognosis of patients who underwent implant-based breast reconstruction after developing local recurrence.

## Patients and methods

### Patient population and study design

We retrospectively evaluated the risk factors for local recurrence and the prognosis of 387 breast cancer patients who underwent implant-based breast reconstruction during the period from 2004 to 2017 in our hospital, after developing local recurrence. We excluded 103 patients, i.e. those with bilateral breast cancer and double cancer, those who underwent secondary reconstruction in our hospital after mastectomy in another hospital, and those with ipsilateral breast tumor recurrence after breast conserving surgery and radiation therapy. Patients with advanced breast cancer receiving post-mastectomy radiation therapy (PMRT) were not in principle considered to be suitable candidates for implant-based breast reconstruction because complications related to PMRT should be avoided and medical treatments must often be delayed.

We defined local recurrence as local treatment failure (local skin, needle biopsy tract, subcutaneous tissue including intramammary lymph node and chest wall), and regional lymph node metastasis was thus excluded. We evaluated risk factors including age (≤ 40 or > 40), magnetic resonance imaging (MRI) findings (segmental lesion; including multiple lesions, or isolated mass), histology (non-invasive carcinoma, invasive carcinoma or other; other histological types and multi-histology lesions), tumor size, nodal status, stage, estrogen receptor (ER), progesterone receptor (PgR) and human epidermal growth factor receptor 2 (HER2) status, subtype, nuclear grade (NG), the presence of extensive intraductal components and lymphatic vessel invasion, the types of surgery and the adjuvant or neoadjuvant treatments (hormonal therapy, chemotherapy and PMRT) for primary lesion administered. If patients had received neoadjuvant chemotherapy, the data obtained prior to this treatment were used. We also evaluated local recurrence free survival and overall survival (OS).

Patients were followed postoperatively by a breast surgeon for 10 years, to monitor for any signs of recurrence. A plastic surgeon then continued follow-up to detect possible breast implant-associated anaplastic large cell lymphoma and implant damage, as well as monitoring the appearance of the reconstructed breast even after the breast surgeon had completed postoperative follow-up.

Our study was approved by institutional review board of Hiroshima City Hospital. Since this was a retrospective study of anonymous data, singed informed consent was waived by the Ethics Committee of Hiroshima City Hospital. In addition, the study was carried out in compliance with the Declaration of Helsinki.

### Surgical approach with plastic surgery team and the concept of reconstruction

During implant-based breast reconstruction in our hospital, mastectomy is performed by breast surgeons and reconstruction is performed by plastic surgeons. A tissue expander is inserted at the same time that mastectomy is performed. The expander is then replaced with an implant approximately 6 months later. Four types of mastectomy are utilized in our hospital: total mastectomy (TM), skin-sparing mastectomy (SSM), nipple-sparing mastectomy (NSM), and areola-sparing mastectomy (ASM). We analyzed TM and SSM together because we have standardised the skin incision line to a spindle shape in both TM and SSM, and the nipple and areola were excised in both mastectomies. We endeavored to make skin incision lines as short as possible. In cases with skin invasion, we extended the skin incision line and removed the skin just above the primary lesion or needle biopsy tract, but if no evidence raising suspicion of skin invasion was present and the judgement was made, based image examinations, that the skin could be preserved, we left the skin just above primary lesion or needle biopsy tract in place to achieve better esthetic results. Moreover, to avoid skin necrosis, we created a slightly thicker mastectomy flap when performing mastectomy with, versus without, breast reconstruction.

ASM is a surgical procedure developed by breast and plastic surgeons at our hospital to enucleate the nipple and preserve the areola, with the aim of reducing nipple recurrence in patients who have received NSM [3], and nipple reconstruction is performed at a later date. We think that ASM is superior from an esthetic perspective because the areola is preserved, and by removing the nipple, where lactiferous ducts converge, it might have the advantage of eliminating the risk of recurrence from the nipple.

### Pathological stratification

As per the American Society of Clinical Oncology (ASCO)/College of American Pathologists (CAP) guidelines, a cut-off level of ≥ 1% nuclear staining of any intensity was used to define a positive ER and/or PgR result. For HER2/neu determination, ASCO/CAP guidelines were followed, with positive results reported in patients with complete intense circumferential membrane staining in > 10% of invasive tumor cells by immunohistochemistry, and/or fluorescence in-situ hybridization showing an HER2/Cep17 ratio of ≥ 2.0 or an average HER2 copy number of ≥ 6 signals per cell. We classified breast cancer into five subtypes: Luminal A-like, Luminal B-like/HER2 negative, Luminal B-like/HER2 positive, HER2 positive, and Triple-negative. NG was determined according to the General Rules for Clinical and Pathological Recording of Breast Cancer, 18th edition [4].

We classified surgical margins into three types: positive, negative and < 2 mm, based on consensus guidelines [5, 6]. According to these guidelines, a negative margin is defined as “no ink on tumor” for invasive carcinoma treated with breast-conserving surgery [5], and “ductal carcinoma in situ (DCIS) within 2 mm from the surgical margin” for DCIS treated with breast-conserving surgery and radiation therapy[6]. The difference in surgical margin classification between these guidelines and the present study is that margin < 2 mm was added, making three types in total including the positive and negative categories. Furthermore, margin < 2 mm was applied not only to non-invasive carcinoma but also invasive carcinoma. The cases lacking detailed records or for which categorization into margin < 2 mm or negative was not possible were deemed “not evaluated” (NE). In patients requiring additional surgical resection after reconstruction because of positive surgical margins, we used the margin results obtained after additional surgical resection.

### Statistical analysis

Results of comparisons between groups were statistically assessed by the chi-square test or Fisher’s exact test. Local recurrence free survival and OS were calculated by the Kaplan–Meier method and comparisons between groups were made using the log-rank test. The risk factors associated with local recurrence were identified using Cox’s proportional hazard regression model. Only the variables identified as statistically significant in the univariate analysis were tested in the multivariate analysis. Adjusted hazard ratios (HR) with 95% confidence intervals (CIs) were reported. Values of *p* less than 0.05 were considered to indicate a statistically significant difference. These analyses were carried out with JMP® 14 (SAS Institute Inc., Cary, NC, USA).

## Results

### Patient characteristics

**Fig. 1:**
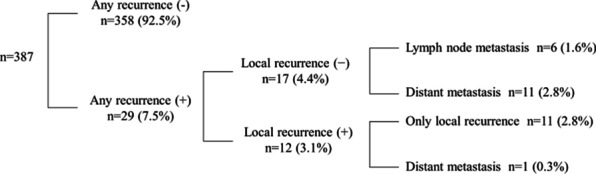
Distribution of patients by recurrence type

The median age was 48 years (range, 21–76), and the median follow-up time was 59 months (range, 7–179). The rate of local recurrence was 3.1% (n = 12). Of 12 patients with local recurrence, 11 had only local recurrence, whereas one also developed distant metastasis (bone and lung metastases). The rate of regional lymph node or distant metastasis, in patients without local recurrence, was 4.4% (n = 17) (Fig. 1).

Table 1 presents a comparison of patient characteristics in the two groups (with and without local recurrence). These data were obtained at the time of implant-based breast reconstruction. Patients younger than 40 accounted for 19.6% (n = 76). Tumor size (T) 1 was most frequent (48.6%, n = 188) and T3 accounted for 4.1% (n = 16) of cases. Patients with lymph node metastasis accounted for 20.2% (n = 78). Early breast cancer (stage 0 or I) and advanced breast cancer (stage II or III) had been diagnosed in 64.9% (n = 251) and 35.1% (n = 136) of patients, respectively. Lymphatic vessel invasion was detected in 33.9% (n = 131) of patients. We performed TM/SSM in 88.9% of patients (n = 344), NSM in 3.6% (n = 14), and ASM in 7.5% (n = 29). The rate of patients with lateral surgical margins that were positive or < 2 mm was 5.2% (n = 20), while the rate of patients with a vertical margin that was positive or < 2 mm was 34.9% (n = 135). PMRT was performed for 3.1% (n = 12) of patients. Four patients with positive surgical margins required additional surgery after reconstruction. Adjuvant hormonal therapy was administered to 71.1% of patients (n = 275) and 32.3% (n = 125) received chemotherapy with or without Trastuzumab.

**Table 1:** Patient characteristics

	Local recurrence				*p* value
	+ (n = 12)		− (n = 375)		
	n	%	n	%	
*Age*					*0.065*
≤ 40	5	41.7	71	18.9	
> 40	7	58.3	304	81.1	
*MRI findings before reconstruction*					0.482
Segmental lesion	11	91.7	350	93.3	
Isolated mass	1	8.3	19	5.1	
NE	0	0.0	6	1.6	
*Histology*					0.408
DCIS/LCIS	3	25.0	84	22.4	
IDC/ILC	5	41.7	266	70.9	
Other	4	33.3	25	6.7	
*Tumor size*					0.470
Is	3	25.0	86	22.9	
1	4	33.3	184	49.1	
2	5	41.7	89	23.7	
3	0	0.0	16	4.3	
*Nodal status*					0.432
0	8	66.7	301	80.3	
1	4	33.3	65	17.3	
2/3	0	0.0	9	2.4	
*Stage*					0.842
0	3	25.0	86	22.9	
I	4	33.3	158	42.1	
II	5	41.7	117	31.2	
III	0	0.0	14	3.7	
*ER*					0.400
+	9	75.0	320	85.3	
−	3	25.0	55	14.7	
*PgR*					0.867
+	9	75.0	289	77.1	
−	3	25.0	86	22.9	
*HER2*					0.453
+	3	25.0	65	17.3	
−	9	75.0	306	81.6	
NE	0	0.0	4	1.1	
*Subtype*					0.552
Luminal A-like	6	50.0	235	62.7	
Luminal B-like HER2 negative	3	25.0	59	15.7	
Luminal B-like HER2 positive/HER2	3	25.0	59	15.7	
Triple Negative	0	0.0	15	4.0	
NE	0	0.0	7	1.9	
*Nuclear grade*					0.402
Low/intermediate/high grade DCIS	3	25.0	70	18.7	
1	3	25.0	154	41.1	
2/3	6	50.0	125	33.3	
NE	0	0.0	26	6.9	
*EIC*					0.981
+	4	33.3	115	30.7	
−	4	33.3	117	31.2	
NE	4	33.3	143	38.1	
*Lymphatic vessel invasion*					0.004
+	9	75.0	122	32.5	
−	3	25.0	247	65.9	
NE	0	0.0	6	1.6	
*Operation*					0.393
TM/SSM	10	83.3	334	89.1	
NSM	1	8.3	13	3.5	
ASM	1	8.3	28	7.5	
*Surgical margin (lateral)*					0.476
+	1	8.3	13	3.5	
< 2 mm	0	0.0	6	1.6	
−	11	91.7	356	94.9	
NE	0	0	0	0	
*Surgical margin (vertical)*					0.007
+	1	8.3	22	5.9	
< 2 mm	8	66.7	104	27.7	
−	1	8.3	170	45.3	
NE	2	16.7	79	21.1	
*Adjuvant PMRT*					1.000
+	0	0.0	12	3.2	
−	12	100.0	363	96.8	
*Neoadjuvant chemotherapy*					1.000
+	0	0.0	21	5.6	
−	12	100.0	354	94.4	
*Adjuvant hormonal therapy*					0.340
+	7	58.3	268	71.5	
−	5	41.7	107	28.5	
*Any chemotherapy* ± *Trastuzumab*					0.535
+	5	41.7	120	32.0	
−	7	58.3	255	68.0	

*MRI*, Magnetic Resonance Imaging; *DCIS*, Ductal carcinoma in situ; *LCIS*, Lobular carcinoma in situ; *IDC*, Invasive ductal carcinoma; *ILC*, Invasive lobular carcinoma; *EIC*, Extensive intraductal component; *TM*, Total mastectomy; *SSM*, Skin-sparing mastectomy; *NSM*, Nipple-sparing mastectomy; *ASM*, Areola-sparing mastectomy; *PMRT*, Post-Mastectomy Radiotherapy

Lymphatic vessel invasion and positive vertical margin rates differed significantly between the two groups (*p* = 0.004 and *p* = 0.007). There was no significant difference in positive lateral margin rates (*p* = 0.476) between the two groups. None of the 12 patients with local recurrence received PMRT.

### Characteristics of patients with local recurrence

Table 2 presents the clinicopathological characteristics of the 12 patients with local recurrence. Early breast cancer (stage 0 or I) was the diagnosis in 5 patients, advanced breast cancer (stage II or III) in 7. The most common reason for detection of local recurrence was subjective symptoms, present in 10 patients, including palpable mass (n = 9) and nipple erosion (n = 1). Two recurrences were detected by imaging (n = 2). Local recurrence sites were residual nipple following NSM (n = 1), subcutaneous tumor around the primary lesion (n = 7), skin just above the primary lesion (n = 2), and the needle biopsy tract (n = 3). All 4 patients with local recurrence involving the skin just above the primary lesion or needle biopsy tract had advanced breast cancer with lymph node metastasis.

**Table 2:** Clinicopathological characteristics, treatments after local recurrence and outcome of patients with local wall recurrence

Patient No	1	2	3	4	5	6	7	8	9	10	11	12
*Clinical characteristics*												
Primary												
Age	51	45	40	37	39	37	48	41	46	40	47	45
Tumor size	Is	Is	Is	1	1	1	1	2	2	2	2	2
Nodal status	0	0	0	0	0	0	0	0	1	1	1	1
Stage	0	0	0	I A	I A	I A	I A	II A	II B	II B	II B	II B
MRI findings	Segmental	Segmental	Segmental	Segmental	Segmental	Segmental	Segmental	Segmental	Isolated mass	Segmental	Segmental	Segmental
Operation	NSM	TM	TM	TM	TM	TM	TM	TM	TM	TM	TM	TM
Adjuvant therapy	–	–	–	HT	HT	–	HT	CT, HT	CT + Trastuzumab	oral CT, HT	CT, HT	CT, HT
Opportunity to detect recurrent lesions	Nipple erosion	Palpation	Imaging inspection	Palpation	Palpation	Palpation	Palpation	Palpation	Palpation	Imaging inspection	Palpation	Palpation
Recurrence												
Local recurrence site												
Needle biopsy tract										+	+	+
Skin just above primary lesion									+	+		
Subcutaneous tumor around primary lesion		+	+	+	+	+	+	+				
Other	Residual nipple											
Other recurrent site	–	–	–	–	–	–	–	–	–	Bone, Lung	–	–
*Pathological characteristics (Primary/Recurrence)*												
Histology	DCIS/Paget's disease with invasion	DCIS/IDC	DCIS/IDC	Mucinous/Mucinous	IDC + ILC/IDC	IDC/lymph node metastasis	IDC/lymph node metastasis	Mucinous/Mucinous	IDC/IDC	IDC/IDC	IDC/IDC	IDC/IDC
Hormone receptor	−/−	+ / +	+ / +	+ / +	+ / +	−/−	+ / +	+ / +	+ / +	+ / +	+ / +	+ / +
HER2	+ / +	−/−	−/−	−/−	−/−	+ / +	−/ +	−/−	+ / +	−/−	−/−	−/−
Ki-67	< 15%/ > 80%	< 15%/ < 15%	< 30%/ < 15% < 15% < 15% < < 15%	< 15%/ < 15%	< 15%/ < 15%	< 80%/ < 50%	< 30%/ < 15% < 15% < 15%	< 30%/ < 15%	< 80%/ < 50%	< 15%/ < 15%	< 15%/ < 15%	> 15%/ < 50% 30,305,050%
Subtype	HER/HER	LA/LA	LA/LA	LA/LA	LA/LA	HER/HER	LB/LH	LB/LA	HER/HER	LA/LA	LA/LA	LB/LB
*Pathological characteristics ( Before/After recurrence)*												
Nuclear grade*	High/3	Low/1	Intermediate/1 11/1	1/1	1/1	3/3	3/3	2/1	3/3	1/1	1/1	2/3
Lymphatic vessel invasion	−/ +	−/−	−/−	±	+ / +	+ / +	+ / +	±	+ / +	+ / +	+ / +	+ / +
Vertical margin (at the time of reconstruction)	Not evaluated	< 2 mm: DCIS	< 2 mm: DCIS	< 2 mm: Invasive	< 2 mm: DCIS	+ : DCIS	< 2 mm: DCIS	< 2 mm: DCIS	< 2 mm: Invasive	Not evaluated	< 2 mm: Invasive	–
Tissues from recurrence site	Residual nipple lactiferous duct	Residual mammary tissue	Residual mammary tissue	Subcutaneous fat	Skin ~ subcutaneous fat	Lymph node	Lymph node	Subcutaneous fat	Skin	Skin ~ subcutaneous fat	Skin ~ subcutaneous fat	Skin ~ subcutaneous fat
*Treatment after recurrence*												
Operation	+	+	+	+	+	+	+	+	+	+	+	+
Radiation	–	+	+	+	–	+	+	+	+	–	+	+
Pharmacological treatment	HT, CT + Trastuzumab	HT	HT	HT	HT	CT + Trastuzumab	HT	HT	T-DM1	HT	HT	CT, HT
*Outcome*												
Local recurrence free survival (M)	155	42	122	19	31	12	39	12	9	24	19	71
Overall survival (M)	184	61	127	97	75	57	57	57	61	71	55	86
Survival	+	+	+	+	+	+	+	+	+	+	+	+

Segmental, segmental lesion; NSM, nipple-sparing mastectomy; TM, total mastectomy; HT, hormonal therapy; CT, chemotherapy; Mucinous, mucinous carcinoma; LA, luminal A-like; LB, luminal B-like/HER2 positive; LH, luminal B-like/HER2 negative; HER, HER2 positive; T-DM1, trastuzumab emtansine; *DCIS: low/intermediate/high grade

As for pathological characteristics, all 3 patients with a diagnosis of DCIS at the time of reconstruction had local recurrence with invasion and all 9 patients with invasive carcinoma at the time of reconstruction had lymphatic vessel invasion. Nine patients had a vertical margin that was positive or < 2 mm at the time of reconstruction (margin-positive DCIS in 1 patient, DCIS < 2 mm in 5 patients, invasive carcinoma < 2 mm in 3 patients). The tissues of origin for the local recurrent lesion included the lactiferous duct in the residual nipple (n = 1), the residual mammary tissue (n = 2), a lymph node (n = 2), and the skin or subcutaneous tissue (n = 7).

### Treatment for local recurrence

Table 2 also shows treatments and outcomes after local recurrence. All 12 patients with local recurrence underwent surgical resection. Nine of the 12 patients also received radiation therapy, the exceptions being one patient with recurrence from the residual nipple following NSM, one with distant metastasis and one who rejected radiation therapy. Radiation was delivered to a permanent implant in all 9 patients, none of whom had complications associated with radiation therapy such as skin necrosis, infection, capsular contracture and so on. With regard to pharmacological therapy, all 4 patients without treatment after reconstruction required pharmacological agent administration. Hormonal therapy was administered to all patients and chemotherapy or anti-HER2 therapy to 4. These treatments resulted in favorable outcomes for the 12 patients, all of whom have remained free of new recurrences to date.

### The cases with remarkable local recurrence (Fig. 2)

**Fig. 2:**
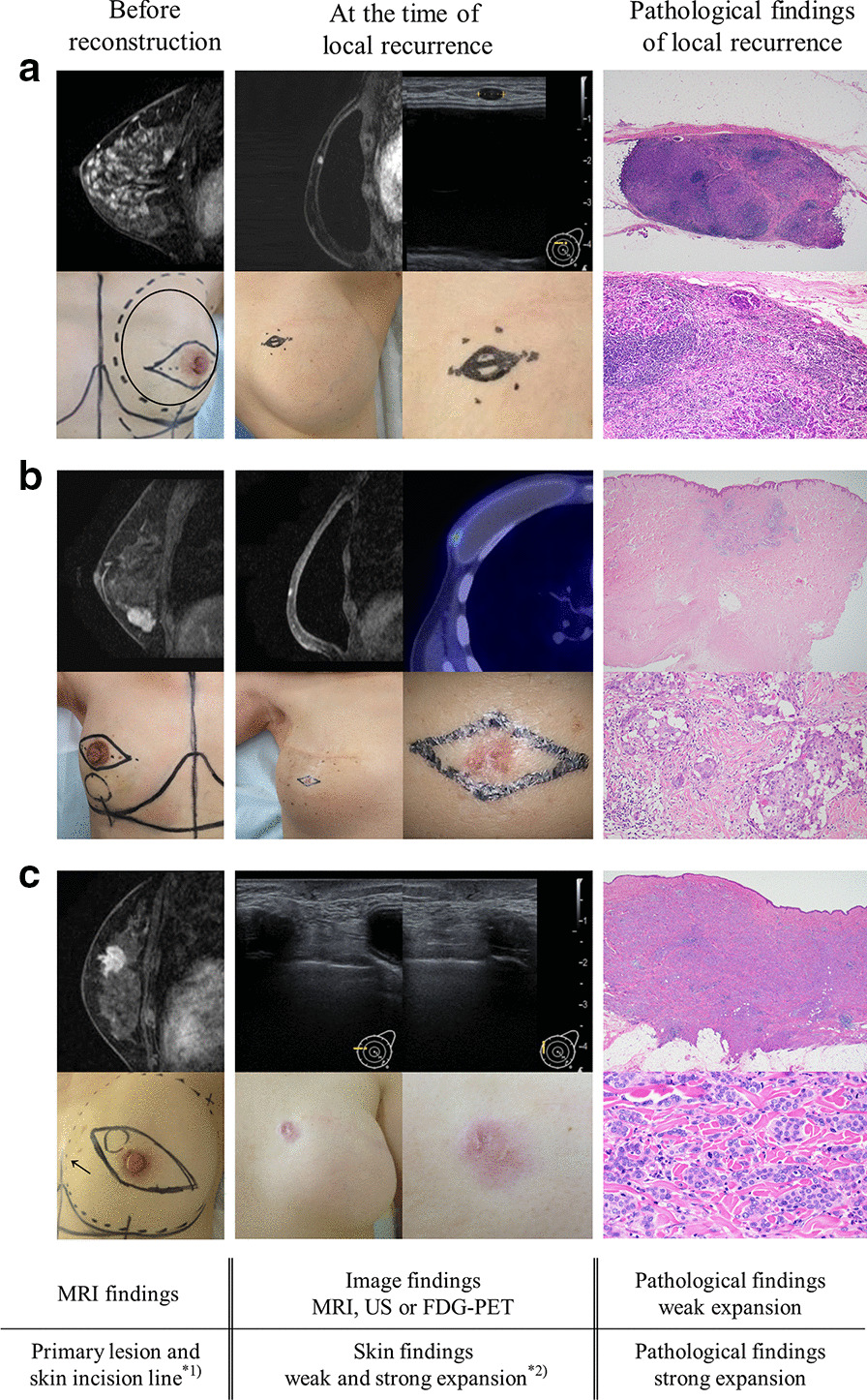
The cases with remarkable local recurrence. **a** Local recurrence from a subcutaneous lymph node. **b** Local recurrence from the skin just above the primary lesion. **c** Local recurrence from needle biopsy tract. *^1)^ circled area (solid line): primary lesion, line corresponding to a spindle shape: skin incision line. ^*2)^ marked area: local recurrence site

(A) Local recurrence from subcutaneous lymph node (Fig. 2A; Table 2, patient No. 7).

Patient No. 7 had stage IA Luminal A-like type breast cancer. Primary lesions showed segmental spread and were close to the skin throughout the breast. Pathological findings at the time of reconstruction showed wide spread DCIS and IDC with lymphatic vessel invasion, and DCIS was in vertical margin < 2 mm. Local recurrence free survival was 39 months. The local recurrence site was detected as a palpable mass and was seen as an isolated mass on MRI and ultrasonography (US), and there were no skin changes. The local recurrence site was a lymph node containing abundant IDC components in pathological finding.

(B) Local recurrence from skin just above primary lesion (Fig. 2b; Table 2, patient No. 9).

Patient No. 9 had stage IIB Luminal B-like HER2 positive type breast cancer. The primary lesion was an isolated mass close to the skin and had not been included in the skin incision line. Pathological findings at the time of reconstruction showed IDC with lymphatic vessel invasion and IDC was in vertical margin < 2 mm. Local recurrence free survival was 9 months. The local recurrence site was an isolated mass on MRI and showed abnormal uptake on FDG-PET. The skin at the recurrence site was erythematous. IDC with lymphatic vessel invasion was recognized in the dermis in pathological finding.

(III) Local recurrence from needle biopsy tract (Fig. 2c; Table 2, patient No. 12).

Patient No. 12 had stage IIB Luminal B-lke HER2 negative type breast cancer. The primary lesion was an isolated mass close to the skin. Segmental lesions showed spreading around the mass. The skin just above the lesion, but not the needle biopsy tract (indicated by the arrow), was included in the skin incision line. Pathological findings at the time of reconstruction showed IDC with lymphatic vessel invasion, but vertical margin was negative. Local recurrence free survival was 71 months. The local recurrence site was detected as a palpable mass and was a low echo area on US. The skin at the needle biopsy tract was erythematous. IDC components were abundant in the skin and subcutaneous tissue and local recurrence was more aggressive than primary lesion in nuclear grade and Ki-67.

### Risk factors for and patient outcomes after local recurrence

On univariate Cox regression analysis, the risk factors for local recurrence following implant-based breast reconstruction were lymphatic vessel invasion (HR, 6.60; 95% CI, 1.78–24.47; *p* = 0.005) and positive or < 2 mm vertical margin (HR, 10.58; 95% CI, 1.32–84.64; *p* = 0.018). Multivariate Cox regression analysis also showed lymphatic vessel invasion (HR, 6.63; 95% CI, 1.40–31.36; *p* = 0.017) and positive or < 2 mm vertical margin (HR, 9.72; 95%CI, 1.23–77.13; *p* = 0.047) to be significant independent risk factors for local recurrence (Table 3).

**Table 3:** Risk factors for local recurrence by Cox proportional hazard regression analysis

Factor	Univariate analysis			Multivariate analysis		
	HR	95% CI	*p* value	HR	95% CI	*p* value
Age			0.069			
≤ 40	2.90	0.92–9.15				
> 40	1.00					
MRI findings			0.520			
Segmental lesion	1.00					
Isolated mass	1.96	0.25–15.37				
Histology			0.423			
DCIS/LCIS	1.00					
IDC/ILC	0.56	0.13–2.34				
Tumor size			0.507			
Is	1.00					
1	0.66	0.15–2.95				
2/3	0.62	0.34–6.13				
Nodal status			0.294			
−	1.00					
+	1.90	0.57–6.32				
Stage			0.849			
0	1.00					
I	0.80	0.18–3.60				
II/III	1.18	0.28–4.97				
ER			0.482			
+	1.00					
−	1.61	0.42–6.04				
PgR			0.965			
+	1.00	0.28–3.44				
−	0.97					
HER2			0.776			
+	1.00					
−	0.82	0.21–3.19				
Subtype			0.526			
Luminal A-like	1.00					
Luminal B-like HER2 negative	2.24	0.55–9.06				
Luminal B-like HER2 positive/HER2	1.56	0.38–6.48				
EIC			0.927			
+	0.94	0.23–3.77				
−	1.00					
Lymphatic vessel invasion			0.005			0.017
+	6.60	1.78–24.47		6.63	1.40–31.36	
−	1.00					
Nuclear grade			0.518			
Low/intermediate/high grade DCIS	1.00					
1	0.75	0.12–4.60				
2/3	1.64	0.33–8.16				
Operation			0.905			
TM/SSM	1.00					
NSM	1.43	0.17–11.88				
ASM	1.44	0.18–11.45				
Surgical margin (lateral)			0.568			
+ , < 2 mm	1.82	0.23–14.24				
−	1.00					
Surgical margin (vertical)			0.018			0.031
+ , < 2 mm	10.58	1.32–84.64		9.72	1.23–77.13	
−	1.00			1.00		
Adjuvant hormonal therapy			0.402			
+	0.61	0.19–1.94				
−	1.00					
Any chemotherapy ± Trastuzumab			0.650			
+	1.31	0.41–4.16				
−	1.00					

The *p*-values less than 0.05 are underlined

*HR*, Hazard ratio; *CI*, Confidence Interval; *MRI*, Magnetic Resonance Imaging; *DCIS*, Ductal carcinoma in situ; *LCIS*, Lobular carcinoma in situ; *IDC*, Invasive ductal carcinoma; *ILC*, Invasive lobular carcinoma; *EIC*, Extensive intraductal component; *TM*: Total mastectomy; *SSM*: Skin-sparing mastectomy; *NSM*: Nipple-sparing mastectomy; *ASM*: Areola-sparing mastectomy; *PMRT*: Post-Mastectomy Radiotherapy

**Fig. 3:**
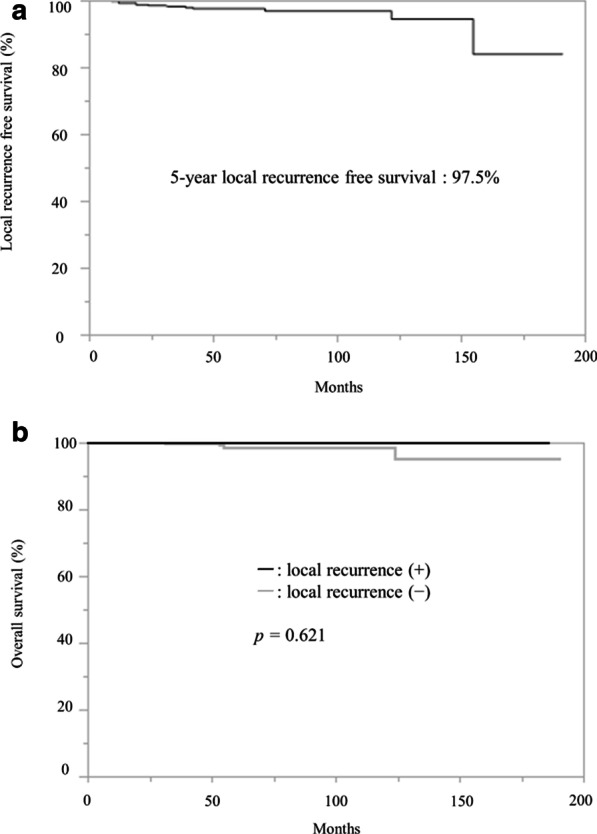
**a** Local recurrence free survival rate for the entire patient population. **b** Overall survival rate according to with or without local recurrence

The 5-year local recurrence free survival rate for the entire patient population was 97.5% (Fig. 3A). The median local recurrence free survival of the 12 patients with local recurrence was 27.5 months (range, 9–71 months). There was no significant difference in OS between patients with versus without local recurrence (p = 0.621) (Fig. 3B).

## Discussion

IBR, including both autologous tissue and implant-based breast reconstructions, is not associated with increases in local recurrence or loco-regional recurrence (LRR) risk [1, 2], and has now become a widely performed surgical procedure. However, due to the short observation period, there are only a few reports on the incidence of LRR following IBR.

In this study, 3.1% of the patients had local recurrence following implant-based breast reconstruction. In reports on LRR following IBR, the LRR rates were 3.2–5.5% [2, 7], similar or slightly higher than the rate in this study. Unlike previous reports, we focused on local recurrence, excluding regional lymph node recurrence, following implant-based breast reconstruction rather than autologous tissue-based breast reconstruction. We excluded lymph node metastasis because it is generally attributed to the malignancy of the breast cancer itself rather than to the implant-based breast reconstruction procedure, and patient selection criteria differ from those for implant-based breast reconstruction in that PMRT can be used in patients who have undergone autologous tissue-based breast reconstruction. We excluded patients expected to need PMRT from the indications for implant-based breast reconstruction because the complications of PMRT should be avoided and adjuvant treatment should not be delayed. In fact, a recent study found that long-term outcomes after implant-based breast reconstruction were impacted by a significantly higher risk of complications requiring reoperation and reconstruction failure after PMRT, as compared to autologous tissue-based breast reconstruction (*p* = 0.004 and *p* = 0.003) [8].

The local recurrence sites were often detected as palpable masses. Another report found the most frequent reason for local recurrence detection to be patient concern leading to examination [9]. This is because an implant was inserted and inflated in a submuscular pocket, facilitating palpation of the recurrence site as a mass. Therefore, it is important to carefully follow-up these patients including physical examination and palpation after implant-based breast reconstruction.

Moreover, local recurrence from skin just above the primary lesion and needle biopsy tract are also highly characteristic of local recurrence. One-third of patients with local recurrence had this type of recurrence and all had advanced breast cancer with lymph node metastasis. In implant-based breast reconstruction, the skin just above the primary lesion and needle biopsy tract sometimes remained due to not being included in the short and small skin incision lines. On the other hand, in performing mastectomy without reconstruction, we usually remove these lesions, such that this type of recurrence does not develop. Moreover, following breast conserving surgery, patients do not develop these types of recurrence. This appears to be attributable to radiation therapy following breast conserving surgery. From the results of this study, removal of the skin just above the primary lesion and the needle biopsy tract might be beneficial in implant-based breast reconstruction, at least in advanced breast cancer cases.

With regard to treatment after local recurrence, all patients with local recurrence underwent surgical resection. As to pathological findings, components of invasive carcinoma were often scattered around the recurrent tumor, and one-quarter of patients required additional surgical resection. It is apparently difficult to accurately assess the extent of recurrence with imaging modalities such as ultrasonography and MRI. An adequate surgical resection margin at the local recurrence site is thus essential. Most patients with local recurrence received radiation therapy delivered to the permanent implant, and none developed complications associated with radiation therapy. Radiation delivered to the tissue-expander is usually not recommended because it often causes complications and reconstruction failure [10–17], but, as previously reported, radiation to the implant does appear to be acceptable [18–22]. Furthermore, we selected pharmacological therapies giving consideration to previous treatments, the degree of malignancy, the time to local recurrence and other relevant factors. These therapies achieved favorable courses, without new recurrence, in all patients with local recurrence.

This study showed patients with lymphatic vessel invasion and vertical margins that were positive or ˂2 mm to be at significantly elevated risk for local recurrence. It was suggested that local recurrence may develop from residual skin or subcutaneous tissue via lymphatic vessel invasion or cancer remaining near the vertical margin. Mastectomy with implant-based breast reconstruction leaves more skin or subcutaneous tissues than mastectomy without reconstruction and in addition, most patients do not receive PMRT after implant-based breast reconstruction. Therefore, the patients with these risk factors may require PMRT. According to other studies, the risk factors for LRR following IBR included certain histopathological subtypes (Luminal B-like/HER2 positive and Triple-negative), body mass index ≥ 25 [7], ER–negative status, age ≤ 40 years, and large tumor (T2 versus T1) [8]. The reason for the difference in risk factors between our and previous studies is that we excluded lymph node metastasis and autologous tissue-based breast reconstruction, as noted above. Importantly, in this study the surgical margins were divided into lateral and vertical margins. Our clinical impression is that patients with primary lesions close to the skin often have a close vertical margin, and these patients are likely to have local recurrence regardless of whether the marginal component is non-invasive or invasive carcinoma. Therefore, we added margin < 2 mm to the positive and negative margin factors, and margin < 2 mm was applied not only to non-invasive carcinoma but also to invasive carcinoma. Our results indicate that when performing implant-based breast reconstruction, it is desirable to evaluate the surgical margins divided into lateral and vertical, and to determine whether the margin is < 2 mm. These criteria appear to be applicable even to cases with invasive carcinoma because, after implant-based reconstruction, patients often do not receive radiation therapy, in contrast to those who have undergone breast conserving surgery.

As for outcomes, the 5-year local recurrence free survival rate for the entire patient population was 97.5% and the median local recurrence free survival of the 12 patients with local recurrence was 27.5 months (range, 9–71 months). 6 patients had local recurrence within 2 years. We need to pay more attention to patients at high risk for local recurrence during postoperative follow-up. The OS did not differ significantly between patients with versus without local recurrence. As demonstrated by another study [23], local recurrence following implant-based reconstruction is generally considered to achieve good outcomes when appropriate treatment is administered.

This study has 3 important limitations. First, the concepts underlying the selection and performance of implant-based breast reconstruction differ among hospitals. We generally excluded advanced breast cancer patients who were expected to need PMRT from among those eligible for receiving implant-based breast reconstruction. However, some hospitals do elect to perform implant-based breast reconstruction for advanced breast cancer patients expected to require PMRT. In addition, approaches to managing patients with surgical margins that are positive or < 2 mm, such as PMRT and additional surgical resection, apparently differ among hospitals. Moreover, the frequency of selecting mastectomy and the thickness of the mastectomy flap also differ from not only among hospitals but also among individual surgeons. Second, it was difficult to evaluate whether the skin just above the primary lesion or the needle biopsy tract had been removed because many patients’ medical records did not include this information. It is hoped that future studies will maintain records pertaining to this issue. Furthermore, one-third of patients with this type of local recurrence had advanced breast cancer, while none of the early breast cancer patients had such recurrences. Therefore, whether the skin just above the primary lesion or the needle biopsy tract should be removed remains unknown, even for patients with early breast cancer. The third limitation is the lack of evaluation of the vertical margin. This is one of the risk factors for local recurrence following implant-based breast reconstruction. However, some of our patients lacked detailed records on the vertical margin. Therefore, we could not determine whether the vertical margin was < 2 mm or negative. Our results indicate the utility of evaluating surgical margins by dividing them into lateral and vertical, with each margin then being further subdivided into positive, negative and < 2 mm.

In conclusion, the local recurrence rate following implant-based breast reconstruction was 3.1%. Physical examination and palpation after implant-based breast reconstruction are important for detecting sites of local recurrence. In advanced breast cancer, it is suggested that removal of the needle biopsy tract and skin just above the primary lesion might be beneficial. The risk factors for local recurrence following implant-based breast reconstruction were lymphatic vessel invasion and a positive or < 2 mm vertical margin. Patients with these risk factors must be carefully followed up and may benefit from receiving PMRT. Patients who develop local recurrence following implant-based reconstruction apparently have good outcomes with appropriate treatment. Implant-based breast reconstruction is increasingly being selected by patients. To provide safe and reliable implant-based breast reconstruction to patients, multicenter trials are needed in the future.

## Data Availability

The datasets analyzed during this study are available from the corresponding author on reasonable request.
